# The search for valved conduit tissue grafts for adults (>22 mm): an ultrasonographic study of jugular vein diameters of horses and cattle

**DOI:** 10.1186/1471-2261-9-38

**Published:** 2009-08-13

**Authors:** Colin C Schwarzwald, Rolf Jenni

**Affiliations:** 1 Internal Medicine, Equine Department, Vetsuisse Faculty, University of Zurich, Winterthurerstrasse 260, 8057 Zurich, Switzerland; 2 Echocardiography, Clinic of Cardiology, Cardiovascular Center, University Hospital Zurich, Zurich, Switzerland

## Abstract

**Background:**

Natural heterologous valved conduits with a diameter greater than 22 mm that can be used for right ventricular outflow tract reconstruction in adults are not commercially available. The purpose of this study was to measure by ultrasonography the maximum diameter of the distended jugular veins of horses and cattle, respectively, to identify a population of animals that would be suitable for post-mortem collection of jugular veins at sizes greater than 22 mm.

**Methods:**

The study population included 60 Warmblood horses, 25 Freiberger horses, 20 Brown Swiss cows, and 20 Holstein cows (including 10 Holstein and 10 Red Holstein). The maximum cross-sectional diameter of the distended jugular veins was measured at a location half-way between the mandibular angle and the thoracic inlet. The thoracic circumference (heart girth length) was used as a surrogate of body size. The jugular vein diameters of the different populations were compared by analysis of variance and the association between heart girth length and jugular vein diameter was determined in each of the four study populations by linear regression analysis.

**Results:**

There was considerable individual variation of jugular vein diameters within each of the four study populations. There was no statistically significant relationship between thoracic circumference and jugular vein diameter in any of the populations. The jugular vein diameters of Brown Swiss cows were significantly larger than those of any of the other populations. Warmblood horses had significantly larger jugular vein diameters compared to Freiberger horses.

**Conclusion:**

The results of this study suggest that the production of bovine or equine xenografts with diameters of greater than 22 mm would be feasible. Differences between species and breeds need to be considered. However, prediction of the jugular vein diameter based on breed and heart girth length in an individual animal is inaccurate.

## Background

Valved conduit tissue grafts are commonly used for right ventricular outflow tract (RVOT) reconstruction in the repair of complex congenital heart defects and for pulmonary valve replacement during the Ross procedure. However, despite intensive experimental and clinical research, the ideal valved conduit has yet to be developed. The availability of suitable pulmonary homografts is limited, especially for urgent procedures. Commonly used xenografts, including porcine aortic valves and valves constructed from bovine pericardium[1], require structural manipulation and lack durability. [2,3] Natural heterologous valved conduits are commonly used as an alternative to homografts and other xenografts, but mid-term outcomes following RVOT reconstruction may be complicated by supravalvular stenosis, excessive intimal peel formation, and severe perigraft scarring. [4-7] Furthermore, current heterologous valved conduits are only available at sizes up to 22 mm diameter, limiting their use to children and young adolescents. Natural heterologous valved conduits with a diameter of greater than 22 mm that could be used for adults are not commercially available to date.

To our knowledge, jugular vein diameters in horses and cattle have not been reported so far. The goal of this study was to measure the maximum diameter of the distended jugular veins by means of ultrasonography in horses and cattle, respectively, and to relate the jugular vein diameters to animal size and breed. The data collected in this study would then allow choosing the animal population that would be most suitable for post-mortem collection of jugular veins at sizes greater than 22 mm.

## Methods

### Study population

Eighty-five horses and 40 cattle were included in the study. The equine population included 60 Warmblood horses (aged 8.5 (4–23) years [median (range)]; 54 castrated males, 6 females) and 25 horses of the Swiss Freiberger breed (aged 4.5 (4–6) years; 14 castrated males, 11 females). The bovine population included 20 Brown Swiss (aged 11 (3–17) years; all females), and 20 Holstein (age 4.5 (2–9) years; all females; including 10 Holstein and 10 Red Holstein). All animals were healthy based on anamnestic information obtained from the owners, and all had normal jugular veins based on inspection, palpation, and ultrasonographic examination. The study was approved by the district veterinary office of the cantons Zurich and Berne, respectively.

### Echocardiographic examinations

All examinations were performed in standing, non-sedated animals gently restrained by an experienced animal handler. A digital ultrasound system (SonoSite MicroMaxx, Siemens Schweiz AG, Zurich, Switzerland) with a multi-frequency linear transducer working at 10 MHz was used. The skin and the hair were wiped off with ketonized ethanol (80%) and coupling gel was applied to ensure adequate contact with the transducer. The maximum cross-sectional diameter of the external jugular vein was measured after 10 to 15 seconds of manual occlusion of the vein at the thoracic inlet. The left and the right jugular vein, respectively, were scanned at a location half-way between the mandibular angle and the thoracic inlet. The measurements were performed parallel to the ultrasound beam at the widest diameter, bisecting the vein into two equal parts. Because the examinations were performed under field conditions and scales were not available, the thoracic circumference (heart girth length, in cm) was used as a surrogate of body size. [8,9] It was measured using a tape measure that was placed around the thorax, immediately behind the olecranon and behind the withers. The measurements were performed at end-expiration, with the animals standing square.

### Data analysis and statistics

Graphical and statistical analyses were performed using commercial computer software (GraphPad Prism v5.01 for Windows, GraphPad Software, San Diego California USA, ). For data analyses, measurements of the left and right jugular vein of each animal were averaged. Summary statistics were performed. Jugular vein diameters of the different populations were compared using a one-way analysis of variance with Tukey's post-hoc test; the 95% confidence intervals of the differences between populations were reported. Linear regression analysis was performed to determine the association between thoracic circumference and jugular vein diameter in each of the four study populations. The level of significance was p < 0.05.

## Results

**Figure 1:**
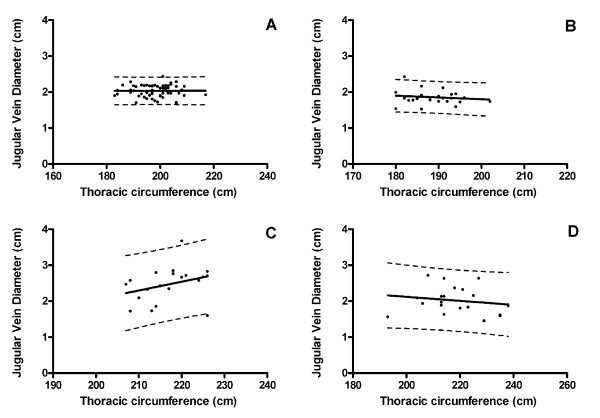
**Relationship between jugular vein diameter and thoracic circumference in Warmblood horses (A), Freiberger horses (B), Brown Swiss cows (C), and Holstein cows (D)**. The solid lines represent the regression lines; the dotted lines represent the 95% prediction band. None of the relationships were statistically significant (Table 1).

The summary statistics and the results of the linear regression analyses are listed in Table 1 and displayed in Figure 1. There was no statistically significant relationship between thoracic circumference and jugular vein diameter in any of the populations, although there was a trend of a positive relationship in Brown Swiss cows. Data analysis showed that there was considerable individual variation within each of the study populations (as indicated by the SD reported in Table 1 and the 95% prediction bands displayed in Figure 1). Table 2 summarizes the results of the comparisons of jugular vein diameters between the four study populations. The jugular vein diameters of Brown Swiss cows were significantly larger than those of any of the other populations (i.e., Holstein cows, Warmblood horses, and Freiberger horses). Warmblood horses had significantly larger jugular vein diameters compared to Freiberger horses.

**Table 1:** Thoracic circumference and jugular diameter of the distended jugular veins in horses and cows of different breeds

**Population**	**Thoracic circumference** **(cm)**	**Jugular vein diameter** **(cm)**		**Linear regression analysis**
			
		**Mean ± SD**	**5%, 95% percentile**	
**Horses**				

Warmblood	198 ± 7	2.03 ± 0.16	1.72, 2.28	p = 0.981

Freiberger	189 ± 6	1.86 ± 0.20	1.53, 2.35	p = 0.389

**Cattle**				

Brown Swiss	217 ± 6	2.47 ± 0.49	1.61, 3.63	p = 0.061

Holstein	218 ± 11	2.02 ± 0.38	1.46, 2.72	p = 0.350

**Table 2:** Summary of the one-way analysis of variance with Tukey's multiple comparison post-hoc test. The level of significance was p < 0.05.

**Comparisons**	**Mean difference (cm)**	**95% CI of difference (cm)**	**Significance**
Brown Swiss vs. Holstein	0.451	0.217 to 0.685	*

Brown Swiss vs. Warmblood	0.437	0.246 to 0.628	*

Brown Swiss vs. Freiberger	0.615	0.393 to 0.837	*

Holstein vs. Warmblood	-0.0140	-0.205 to 0.177	ns

Holstein vs. Freiberger	0.164	-0.0581 to 0.386	ns

Warmblood vs. Freiberger	0.178	0.00183 to 0.355	*

CI, confidence interval; ns, not significant.

## Discussion

The results of this investigation provide information on the *in vivo *diameter of the distended jugular veins, determined by ultrasonography, in four different equine and bovine populations.

The jugular vein diameters of Brown Swiss cows were in agreement with a previous investigation, in which the diameter of distended jugular veins, determined by ultrasonography, was found to be 2.4 ± 0.23 cm. [10] To our knowledge, jugular vein diameters of other bovine breeds and of horses have not been reported to date.

Based on the results of this study, venous diameters of up to 2.4 cm can be expected in a population of average-sized Warmblood and Freiberger horses, with slightly larger veins found in Warmblood horses. At the time of the investigation, very large horses (i.e., estimated body weight above 600 kg) were not available for examination. While direct extrapolation of these findings to a population of larger horses is not possible, diameters greater than 2.4 cm may well be found in draft breed horses. In this study, Brown Swiss cows had the largest jugular vein diameters, exceeding those of Holstein cows and horses, respectively. Jugular veins with a diameter greater than 3 cm can be readily found in Brown Swiss cows.

The use of the heart girth length as a surrogate of body weight may be considered a limitation of this study. However, heart girth length has been shown to correlate fairly well with body weight in horses and cattle. [8,9] Furthermore, weight estimation using body measurements is a simple, practical approach that can be easily used under field conditions, when scales are not readily available.

## Conclusion

In conclusion, the range of jugular vein diameters found in this study suggests that the production of bovine or equine xenografts with diameters of greater than 22 mm would be feasible. Differences between species and breeds need to be considered. However, within each population (i.e., species and breed), there was no significant relationship between jugular vein diameter and body size estimated by girth length, and the range of jugular vein diameters varied considerably. Therefore, prediction of the jugular vein diameter in an individual animal based on breed and girth length is inaccurate.

## Competing interests

The authors declare that they have no competing interests.

## Authors' contributions

CCS was responsible for planning and conducting the study, for data analysis and statistics, and for manuscript writing. RJ initiated the study, contributed to the study design, helped with data collection, and assisted with manuscript writing. Both authors read and approved the final manuscript.

## Authors' information

CCS: Dr. med. vet., PhD, Dipl. ACVIM, Senior Lecturer. RJ: MD, MSEE, Professor.

## Pre-publication history

The pre-publication history for this paper can be accessed here:


